# Molecular Regulation of Betulinic Acid on α3β4 Nicotinic Acetylcholine Receptors

**DOI:** 10.3390/molecules26092659

**Published:** 2021-05-01

**Authors:** Shinhui Lee, Woog Jung, Sanung Eom, Hye Duck Yeom, Heui-Dong Park, Junho H. Lee

**Affiliations:** 1 Department of Biotechnology, Chonnam National University, Gwangju 61186, Korea; dltlstn39@gmail.com (S.L.); yeomself2355@gmail.com (S.E.); 2 School of Food Science and Biotechnology, Kyungpook National University, Daegu 41566, Korea; crazycell79@nate.com; 3 GoPath Laboratories, Buffalo Grove, IL 60089, USA; hyeduck@gmail.com

**Keywords:** α3β4 nAChR, betulinic acid, insomnia, sleep regulation, TEVC, Zizyphus seed

## Abstract

Betulinic acid (BA) is a major constituent of Zizyphus seeds that have been long used as therapeutic agents for sleep-related issues in Asia. BA is a pentacyclic triterpenoid. It also possesses various anti-cancer and anti-inflammatory effects. Current commercially available sleep aids typically use GABAergic regulation, for which many studies are being actively conducted. However, few studies have focused on acetylcholine receptors that regulate wakefulness. In this study, we utilized BA as an antagonist of α3β4 nicotinic acetylcholine receptors (α3β4 nAChRs) known to regulate rapid-eye-movement (REM) sleep and wakefulness. Effects of BA on α3β4 nAChRs were concentration-dependent, reversible, voltage-independent, and non-competitive. Site-directed mutagenesis and molecular-docking studies confirmed the binding of BA at the molecular level and showed that the α3 subunit L257 and the β4 subunit I263 residues affected BA binding. These data demonstrate that BA can bind to a binding site different from the site for the receptor’s ligand, acetylcholine (ACh). This suggests that BA may be an effective antagonist that is unaffected by large amounts of ACh released during wakefulness and REM sleep. Based on the above experimental results, BA is likely to be a therapeutically useful sleep aid and sedative.

## 1. Introduction

Zizyphus plants belong to the family of Jujube. Its seeds have been used throughout history in traditional Asian medicine, mainly as therapeutic agents for insomnia and anxiety [1]. Its seed extract has anxiety-reducing and sedative effects related to sleep time [2]. Zizyphus seed extract can also stimulate the expression of neurotrophic constituents to increase growth and survival of nerve cells in the central nervous system [3]. While the underlying mechanisms of sleep disorders such as insomnia are not fully understood yet, it is generally agreed by researchers that brain activities during sleep patterns are related to such disorders [2]. To date, sleep lengthening effects through GABAergic activity regulation [4] have been well studied. However, wakefulness-related acetylcholinergic mechanisms remain to be elucidated. Thus, this study focused on effects of Zizyphus seeds on acetylcholine receptors.

Nicotinic acetylcholine receptors (nAChRs) vary in structure and function depending on the combination of various subunits [5]. The α3β4 nicotinic acetylcholine receptor (α3β4 nAChR), the focus of the present study, is a neuronal type receptor [6] with a pentamer structure composed of five protein subunits that can mediate synaptic transmission between muscle cells and acetylcholine receptors expressed [7,8]. γ-aminobutyric acid (GABA) is a major inhibitory neurotransmitter in the vertebrate brain that can suppress nerve cell activity. It is modulated in some capacity by most sedative and hypnotic therapeutics. Acetylcholine (ACh), on the other hand, can increase the activity of neurons in the brain and regulate wakefulness. Acetylcholine receptors are divided into two types, muscarinic and cholinergic. It has been shown that cholinergic receptors can regulate rapid-eye-movement (REM) sleep at the molecular level [9]. Previous studies have also shown that while acetylcholine release is reduced during a non-rapid-eye-movement (NREM) sleep, it is greatly increased during an REM sleep [10]. As increased ACh is required for REM sleep, most current sedative and hypnotic drugs can reduce the REM sleep phase and promote the NREM sleep phase [4].

Sleep regulation is related to multiple neurotransmitters including dopamine, serotonin, GABA, and glutamate [11]. Typically, GABA and adenosine are associated with sleep while acetylcholine, histamine, and serotonin are associated with wakefulness. Most sleep-treating drugs currently approved by the Food and Drug Administration (FDA) regulate GABA or melatonin receptors. Although rare, drugs that control histamine are also present [12].

Betulinic acid (3β, hydroxy-lup-20 (29) -en-28-oic acid), one of components of Zizyphus seeds, is a lupane-type pentacyclic triterpenoid [13] that is known for its anti-viral and anti-cancer effects. Triterpenoids are products biosynthesized by some plants as secondary metabolites. They have been used medicinally throughout history [14]. They are composed of 30 carbon atoms that are polymerized to form six isoprene units. They exist in the form of a free state or combination with sugars forming glycosides and esters [15]. As natural products with obvious therapeutic value found abundantly in many plants, triterpenoids have been actively studied as potential therapeutic agents [16,17]. Numerous natural products have been found to be beneficial to human health. Proper investigations of actions of natural products have led to the development of many new drugs in the past. Research on betulinic acid as a new sleep-regulating agent suggests that utilizing natural products existing in plants will be meaningful.

The objective of the present study was to investigate the role of betulinic acid in regulating acetylcholine receptors. After α3β4 nAChRs mRNA was injected into *Xenopus* oocytes and expressed, ACh and BA-induced inward currents were recorded using a two-electrode voltage clamp (TEVC). Our results showed that betulinic acid had an inhibitory effect on α3β4 nAChRs and that such effect was non-competitive with the endogenous ligand. Additionally, we found that inhibitory effects of BA were concentration-dependent, voltage-independent, and reversible. As AChRs in the brain are vital for the regulation of REM sleep, the inhibitory activity of betulinic acid suggests that BA might have potential as a therapeutic hypnotic agent.

## 2. Results

### 2.1. Concentration-Dependent Inhibitory Effect of BA on α3β4 nAChRs

**Figure 1:**
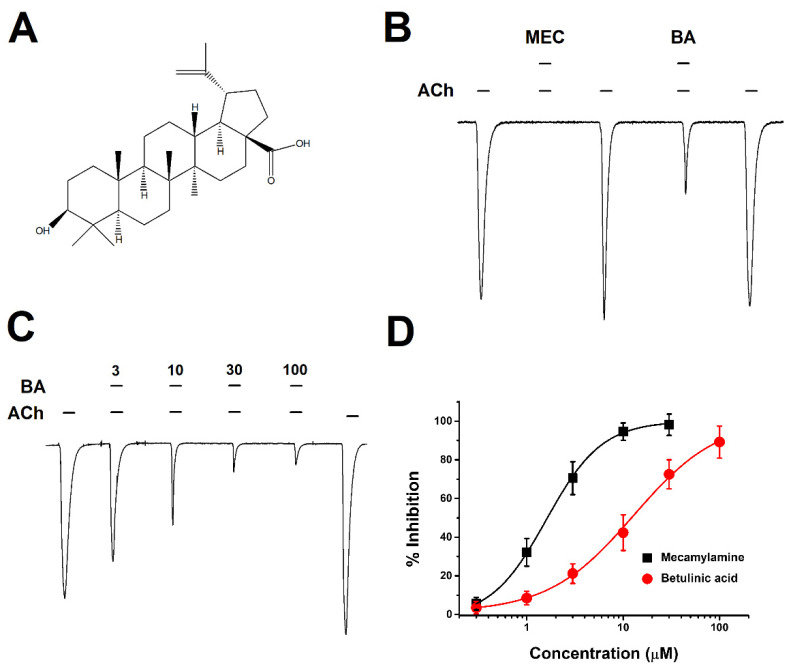
Chemical structure of betulinic acid (BA) and activation manner on bovine α3β4 nicotinic acetylcholine receptors expressed in *Xenopus* oocytes. (**A**) Structure of BA. (**B**) Inhibition of I_ACh_ on bovine α3β4 nAChRs by mecamylamine (MEC), a nicotinic acetylcholine receptor antagonist, and BA. ACh, MEC, and BA were administered at 2 mL per min. The applied concentration of ACh was 100 μM. MEC and BA were administered at 10 μM. The trace indicated inward current in the result of a co-applied and induced reversible inward current (*n* = 6–8 from four different frogs). (**C**) Concentration-relationship results induced by co-treatment with ACh and BA for α3β4 nAChRs. The applied ACh concentration was fixed at 100 μM (*n* = 8–10 from five different frogs). (**D**) The inward current induced by the concentration-relationship of BA and MEC. The inhibition percentage curve of BA and MEC in α3β4 nAChRs was fitted with the Hill equation. Each point represents the mean ± SEM (*n* = 7–9/group). The oocyte voltage-clamp holding potential was −80 mV.

An electrophysiology experiment using a two-electrode voltage clamp was performed to examine the effect of BA on α3β4 nicotinic acetylcholine receptors (α3β4 nAChRs). At one minute after administration of 2 mL of 100 μM acetylcholine (ACh) an inward current appeared from oocytes injected with α3β4 nAChRs. The primary binding site for α3β4 nAChs is between α and β subunit [18]. Based on this fact, it was confirmed that correct expression and proper assembly of the receptor were achieved in Figure 1B. When mecamylamine (MEC), a nicotinic acetylcholine receptor antagonist, was added at the same time as ACh, we found nearly 100% inhibition of I_ACh_ (acetylcholine-induced inward peak current). BA co-administered with ACh also showed inhibition similar to MEC, an antagonist of AChR. When ACh was again added, the inward current fully recovered, confirming that effects of BA were reversible. Additionally, inhibitory effects of BA were concentration-dependent in Figure 1C. When the concentration of BA was increased, ACh-induced inward current was decreased. When BA was applied at the same concentration of 100 μM as ACh, the inhibition percentage of nAChRs by BA was 89.2 ± 8.2%. In Figure 1D, compared to the inhibition manner of MEC that was affected by concentration, BA inhibited the current in a similar manner but at a different degree. IC_50_ (half-maximal inhibitory response concentration) for BA was at 12.8 ± 2.0 μM, higher than that of MEC at 1.5 μM. Thus, although their efficacies were similar, their potencies were different. The Hill coefficient was 0.9 ± 0.1 for BA and 1.5 ± 0.1 for MEC. These data showed that the inhibition of α3β4 nAChRs by BA was concentration-dependent.

### 2.2. Current-Voltage Relationship of I_ACh_ and Non-Competitive Inhibition by Betulinic Acid

**Figure 2:**
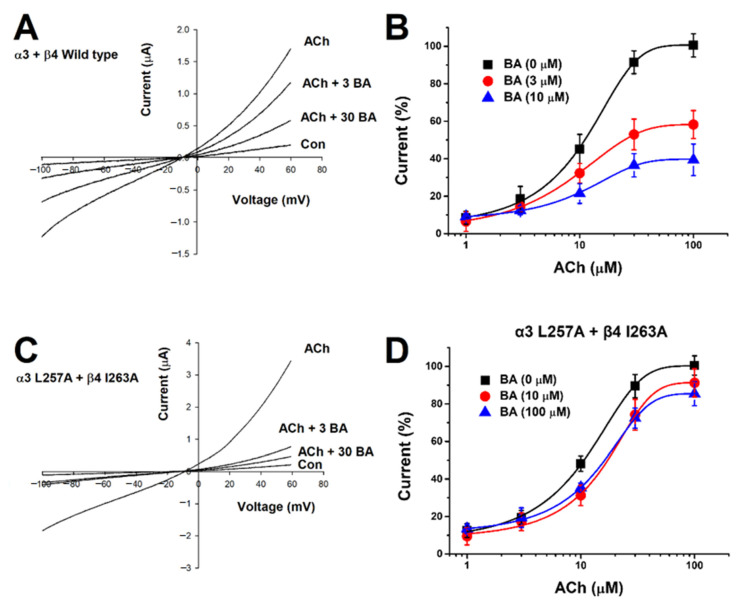
Confirmation of α3β4 nAChRs current-voltage relationship and non-competitive action of BA. (**A**) Confirmation of the interaction between the response of α3β4 nAChRs and different concentrations of BA using a voltage ramp protocol from −100 to +60 mV. The membrane holding potential was −80 mV. The applied ACh concentration was fixed at 100 μM (*n* = 8–10 from four different frogs). (**B**) After applying various concentrations of ACh, induced internal current and non-competitive action of BA were observed. The concentration of BA applied was 3 μM (●) or 10 μM (▲). The holding potential of the oocyte voltage-clamp was −80 mV. Each point represents mean ± SEM (*n* = 7–9/group). (**C**) Double-mutant α3β4 nAChRs (α3: L257A, β4: I263A) and the application of different concentrations of BA using a voltage ramp protocol from −100 to +60 mV. The membrane holding potential was −80 mV. The applied ACh concentration was fixed at 100 μM (*n* = 7–9 from five different frogs). (**D**) Applying various concentrations of ACh and BA to double-mutant α3β4 nAChRs (α3: L257A, β4: I263A). The concentration of BA applied was 10 μM (●) or 100 μM (▲). The holding potential of the oocyte voltage-clamp was −80 mV. Each point represents mean ± SEM (*n* = 7–9/group).

Current-voltage relationship about the inhibition of α3β4 nAChR expressing oocytes with BA was determined. The experiment was conducted with a ramp protocol and the holding potential was set between −100 mV and +60 mV. The membrane potential was held in an artificially controlled state at −80 mV using a current electrode. In Figure 2A, when ACh and BA were co-administered, the reversible potential approached about −10 mV. When the inward-current caused by voltage fluctuation (−100 to +60 mV) was converted into a slope, we found that the slope was not affected by voltage. These data suggest that α3β4 nAChRs are voltage-independent. In Figure 2B, to further explore mechanisms of BA inhibition, different concentrations of ACh were co-administered with fixed concentrations of BA (3 μM or 10 μM) in oocytes expressing α3β4 nAChRs. As the concentration of BA increased, the EC_50_ of ACh changed and the efficacy was decreased. This presents a non-competitive manner. In the absence of BA, the EC_50_ of ACh to α3β AChR was 9.4 ± 1.25 µM and the maximum efficacies of ACh-induced inward current (E_max_) were 100.65 ± 1.47%, 58.24 ± 1.05%, and 39.74 ± 1.39% at BA of 0, 3, and 10 µM, respectively. The dose response-curve was affected by the concentration of applied BA. To summarize, the efficacy of acetylcholine-induced inward current was decreased about 60% by 10 μM BA compared to that by 0 μM BA. These data support that BA works in a non-competitive manner on α3β4 nAChRs.

### 2.3. Docking Model of Betulinic Acid and Nicotinic Acetylcholine Receptor α3β4

**Figure 3:**
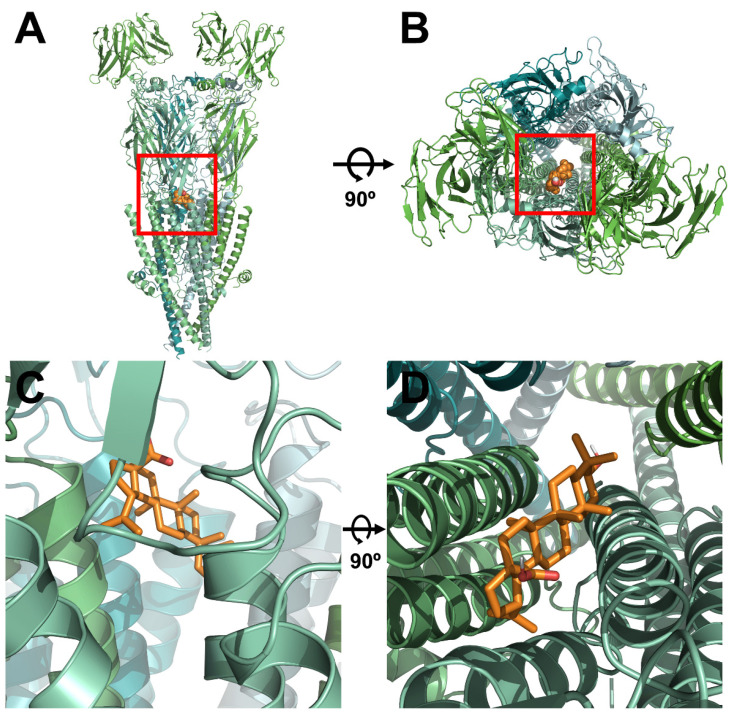
Molecular interaction of BA based on the docking model of α3β4 nAChRs in 3D structure. (**A**–**D**) A view of the BA interaction site on α3β4 nAChRs from the side and top. This structure was constructed based on the structure shown in the protein data bank (PDB) (ID 5T90).

**Figure 4:**
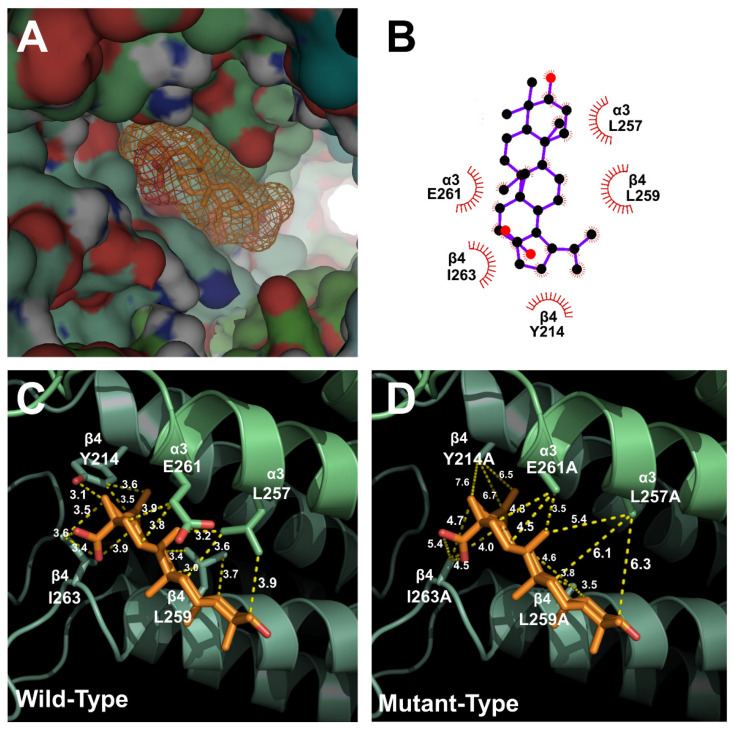
Molecular docking model of BA with wild-type and double-mutant α3β4 nAChRs. (**A**) Binding pocket site in the transmembrane domain of α3β4 nAChRs. BA docked to the transmembrane domain close to the ion-permeable pore. (**B**) The predicted binding site of BA in a 2D schematic. (**C**) The binding interaction energy of BA and residues in wild-type α3β4 nAChRs. (**D**) The binding interaction energy of BA and residues in five mutant channels of α3β4 nAChRs.

To determine the relationship between the binding site of ACh and BA, α3β4 nAChRs were mutated. Wild-type and mutant α3β4 nAChRs were then compared using a covalent docking model. Through a best-fit analysis, the most probable binding position in the receptor’s 3D structure was identified. In Figure 3, the BA binding position was located in the upper part of the transmembrane (TM) domain between α3 and β4 subunits. This position was close to the ion-permeable pore. Through in silico modeling, it was confirmed that the optimal docking site strongly interacted with the BA binding part of wild-type α3β4 nAChRs. Autodock 4.0 program was used to confirm the activity of each residue and derive the highest probability binding sites of the most stable structure. To confirm the interaction between each residue of α3β4 nAChRs and BA, the interaction distance was calculated for mutant α3β4 nAChRs in which each residue was replaced with an alanine residue. In Figure 4C, for wild-type α3β4 nAChRs, BA interacted with the following five residues: α3 subunit (E261; distance = 3.9, 3.2 and 3.8 Å), α3 subunit (L257; distance = 3.2, 3.6 and 3.9 Å), β4 subunit (Y214; distance = 3.1, 3.5 and 3.6 Å), β4 subunit (L259; distance = 3.0, 3.7, and 3.4 Å), and β4 subunit (I263; distance = 3.6, 3.4, 3.5 and 3.9 Å). In Figure 4D, for double-mutant α3β4 nAChRs (α3: L257A, β4: I263A), BA interacted with the following residues: α3 subunit (E261A; distance = 4.3, 3.5, and 4.5 Å), α3 subunit (L257A; distance = 5.4, 6.1 and 6.3 Å), β4 subunit (Y214A; distance = 7.6, 6.7 and 6.5 Å), β4 subunit (L259A; distance = 4.6, 3.8 and 3.5 Å), and β4 subunit (I263A; distance = 5.4, 4.5, 4.7, and 4.0 Å). It was confirmed that BA interacted with two residues of α3 subunit and three residues of β4 subunit. The molecular interaction image and each distance are displayed in Figure 3 and Figure 4.

### 2.4. Inhibitory Effect of Betulinic Acid on a Double-Mutant Nicotinic Acetylcholine Receptor α3β4

**Figure 5:**
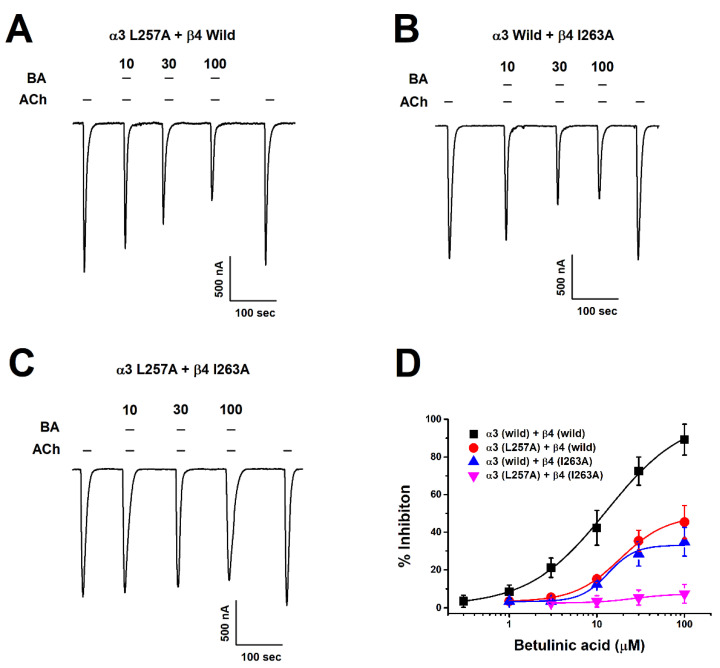
Inhibition of BA by α3β4 nAChRs mutant types. (**A**–**C**) Concentration-response of inward current depending on mutant type. ACh and BA were co-applied. The applied ACh concentration was fixed at 100 μM while BA concentrations included 10, 30, and 100 μM (*n* = 7–10 from four different frogs). (**D**) Graph represents the inhibition of BA activity by mutant type at various concentrations. Each point is presented as mean ± SEM (*n* = 7–9/group). Detailed values are shown in Table 1.

After setting the binding site of BA in silico, we acquired mutant genes via point mutation and determined changes in inhibitory effects of BA on wild-type and mutant-type α3β4 nAChRs through TEVC. We found that BA did bind all mutant genotypes, including one-subunit mutant α3 (L257A) + wild β4 (Figure 5A) and wild α3 + β4 (I263A) (Figure 5B). In Figure 5C, the combined double-mutant α3β4 (α3: L257A, β4: I263A) showed a significantly reduced inhibition compare to one-subunit mutants. In Figure 5D, IC_50_ value varied by mutant. The single mutant α3 (L257A) + wild β4 had an IC_50_ of 18.64 ± 1.71 μM. The wild α3 + β4 (I263A) had an IC_50_ of about 13.4 ± 1.97 μM and the double-mutant α3β4 (α3: L257A, β4: I263A) had an IC_50_ of about 26.2 μM. In Figure 2C, when acetylcholine and BA were co-administered to the double-mutant α3β4 nAChR (α3 L257A + β4 I263A), results were similar to wild-type receptors, showing that voltage was largely unaffected. In Figure 2D, in the case of the double-mutant α3β4 nAChR (α3 L257A + β4 I263A), the inhibitory activity of BA-induced current was significantly offset. Regarding the inhibition of wild-type α3β4 nAChRs by BA, the IC_50_ was 12.89 ± 2.07 μM and I_max_ was 101.59 ± 5.98%. For the inhibition of double mutant-type α3β4 nAChRs (L257A + I263A) by BA, the IC_50_ was 26.23 μM and the I_max_ was 7.4%. As the mutant type affected the binding of BA, the I_max_ was significantly decreased. These data indicate that BA-induced inhibition of α3β4 nAChRs activity is highly associated with the L257 residue of the α3 subunit and the I263 residue of the β4 subunit. Table 1 presents half-inhibitory concentration (IC_50_), Hill coefficient (n_H_), and I_max_ for wild-type and mutant α3β4 nAChRs.

## 3. Discussion

Triterpenoids have anticancer effects by regulating both apoptotic and cell growth-promoting pathways [19]. Betulinic acid (BA), one of pentacyclic triterpenoids, has been previously shown to have anticancer effects mediated by the mitochondrial apoptosis pathway in cancer cells [14].

Our results suggest that BA may reduce rapid-eye-movement (REM) sleep through a molecular interaction with cholinergic α3β4 nAChRs, thus regulating wakefulness of mammals. The release of acetylcholine (ACh) is abundant during REM sleep and wakefulness [20]. One study has shown that the cholinergic receptor antagonism can block the increase in arousal and the decrease in slow-wave or non-rapid-eye-movement (NREM) sleep caused by the regulation of cholinergic neurons [21]. Therefore, the receptor’s antagonist could potentially reduce the arousal stage and the REM sleep phase by regulating cholinergic neurons.

Acetylcholinergic neuron activity is elevated in electroencephalograms (EEG) during wakefulness [22]. Additionally, the release of acetylcholine in the peri-locus coeruleus α (LCα) is increased before the REM sleep phase. This pre-REM ACh release is also associated with cholinergic neurons. Previous research has suggested that neurons in the pedunculopontine tegmentum (PPT) can regulate ACh release prior to and during REM sleep [23]. Therefore, ACh activity is closely associated with REM sleep and wakefulness.

In this study, we confirmed the I_ACh_ was affected when BA was administered to oocytes that expressed α3β4 nAChRs. Simultaneous processing of BA and ACh inhibited ACh-induced inward current in oocytes. This inhibition was concentration-dependent, reversible, voltage-independent, and non-competitive, suggesting that this inhibition occurred by binding to a site separate from the ACh binding site. We hypothesize that the binding of BA can induce a conformational change in α3β4 nAChRs in a non-competitive manner, thereby reducing the wakefulness typically generated by ACh through normal neuronal regulation. Because of its non-competitive action, BA most likely binds to an allosteric ligand-binding site as opposed to a specific ACh binding site. It is also possible that BA can trigger an inhibition without causing conformational changes of the receptor.

Our data also showed that various mutations of α3β4 nAChRs’ subunits could affect BA binding. Through an initial docking study and site-directed mutagenesis, we confirmed that L257 of the α3 subunit and I263 of the β4 subunit affected BA binding. L257 and I263 residues were proposed as BA binding sites because when these residues were changed to alanine, such change did not affect the binding of ACh, although it did affect the binding of BA.

Results of previous studies have revealed that Zizyphus seed compounds can protect nerve cells and increase cAMP levels in the plasma and hippocampus after oral administration of Zizyphus seed in vivo [3]. It has been suggested that Zizyphus seed compounds can pass through the blood-brain barrier (BBB) [3]. Sleep patterns are associated with endogenous circadian rhythms that regulate BBB permeability. These compounds are permeable to the BBB [24]. As the ability of a compound to penetrate the CNS is rare, the fact that these compounds can bypass the BBB cannot be understated. Many potentially useful therapeutics have failed during development because they are unable to effectively reach the CNS. Penetration into the CNS is influenced by (1) passive transport which is mediated by the molecule’s lipophilic characteristics, (2) active transport which is very rarely displayed in polar molecules, and (3) local hydrophobicity [25,26]. The permeability of BBB varies depending on the triterpene group. Although the saponin group cannot pass the BBB, the lupane-type triterpene is lipophilic [18]. It has been demonstrated that lupine-type triterpene can penetrate the BBB [27]. Lupane-type triterpenoids have (1) lupeol, (2) betulin, and (3) betulinic acid. Each lupane-type triterpenoid differs depending on what is attached to the side chain. Betulinic acid has a carboxylic acid at the C-28 position [28]. As this structure is relatively lipophilic, it is likely to have a passive transport that allows BA to penetrate into the CNS [25]. Therefore, a lupane-type triterpenoid BA can permeate BBB. It is presumed that it has a regulatory effect on cholinergic α3β4 nAChRs that exist in CNS to prolong sleep time through a non-competitive binding.

In summary, BA, a pentacyclic triterpenoid, seems to be able to act as an antagonist of α3β4 nAChRs that regulate wakefulness and REM sleep. One study has shown that cholinergic neuron antagonists could block the activity of nAChRs and the increase of wakefulness-related state by cholinergic stimulation [21]. ACh receptors have multiple binding sites for agonist and non-competitive antagonists [6]. The release of the neurotransmitter acetylcholine is much greater during REM sleep [29]. Taken together, our data show that BA is an effective non-competitive antagonist that may not be affected by released AChs during REM sleep or wakefulness. In this experiment, inhibition of α3β4 nAChRs by BA was confirmed. However, verification using various cholinergic nAChRs (expressed in CNS) needs to be performed in the future. BA as a molecule that regulates various cholinergic receptors important for sleep regulation should be verified by further studies. These results suggest that BA, one of components of Zizyphus seed used throughout the history of Asian medicine as a sedative, might be a useful agent to regulate wakefulness and sleep.

## 4. Materials and Methods

### 4.1. Materials

Bovine cholinergic receptor nicotinic α3 subunit (gene accession number: 282702) and β4 subunit (gene accession number: 282181) were obtained from OriGene (Rockville, State Abbr. USA). All reagents, including ACh, MEC, and BA, were purchased from Sigma-Aldrich (St. Louis, MO, USA). All reagent stocks used in electrophysiological experiments were dissolved in dimethyl sulfoxide (DMSO) with the final concentration of DMSO not exceeding 0.01%. Frogs (*Xenopus laevis*) used in these experiments were obtained from the Korean Xenopus Resource Center for Research (KXRCR000001) and used according to appropriate standards.

### 4.2. Oocyte Preparation

For *Xenopus laevis* used in the experiment, the total number of surgeries did not exceed four per head. All experiments were performed according to appropriate standards. After anesthetizing frogs on ice preoperatively, oocytes were extracted. Outer layers of oocytes were removed with OR-2 (Oocyte Ringer 2 solution) (82.5 mM NaCl, 2 mM KCl, 1 mM MgCl2, 5 mM HEPES, pH 7.4) containing collagenase II (2 mg/mL) in a room temperature shaker. Only stage V–VI oocytes [30] were selected and used in this study. These oocytes were incubated in ND96 incubation buffer (58.44 mM NaCl, 2 mM KCl, 1 mM MgCl2, 1.8 mM CaCl2, 5 mM HEPES, 2.5 mM sodium pyruvate, and 50 mg/mL gentamycin, pH 7.4) at 18 °C prior to mRNA injection.

### 4.3. In Vitro Transcription of mRNA and Injection into Oocytes

Bovine α3β4 nAChRs cDNAs were restricted using appropriate restriction sites. Bovine α3β4 nAChRs mRNA was then subjected to in vitro transcription using a T7 polymerase kit (mMESSAGE mMACHINE T7 Transcription Kit; Thermo Fisher Scientific, Waltham, MA, USA) followed by injection into oocytes. A glass needle filled with mineral oil was used to install onto a nanoinjector (Drummond Scientific, Broomall, PA, USA). After incubating for 1–3 days, mRNA-injected oocytes were used for electrophysiology experiments.

### 4.4. Mutagenesis

Using α3 and β4 subunit cDNAs, point mutation was performed by PCR with Pfu DNA polymerase (Quikchange II Site-Directed Mutagenesis Kit). After designing a chimeric primer for mutation, optimal PCR conditions were determined. DpnI was added to remove methylated cDNA. Mutated DNA was transformed into a pGEM vector. All acquisition mutated DNAs were confirmed for sequencing to ensure that they were composed of appropriate sequences. DNA sequencing was performed by Cosmo Gentech Inc (City, State Abbr. Country).

### 4.5. D Structure Modeling and Molecular-Docking Studies

Using a molecular-docking software Autodock (version 1.5.6), the binding site of BA in α3β4 nAChRs was determined. Protein structure of α3β4 nAChRs was obtained from the Protein Data Bank (ID code5T90). The 3D structure of BA was constructed based on the molecular structure presented by PubChem. Modeling of protein-ligand interactions was implemented using Autodock and Ligplot considering intermolecular energy and suppression constants. The resulting complex structure was analyzed using EMBL-EBI and Pymol by Schrödinger with Ligplot (version 4.5.3). Amino acid distance of α3β4 nAChRs wild-type and mutant interacting with BA was determined by Pymol.

### 4.6. Data Recording with Two-Electrode Voltage Clamp

Two-electrode voltage clamp (TEVC) experiments were performed using oocytes infused with α3β4 nAChR mRNA and cultured according to appropriate criteria. Each oocyte was penetrated with a microelectrode filled with 3M KCl at a resistance of 0.2 MΩ. All experiments were performed at room temperature using a two-electrode voltage clamp (OC-725C; Warner Instruments, Hamden, CT, USA) with a Digidata 1322A. The holding potential of the oocyte membrane potential was −80 mV. ACh and BA used in the experiment were diluted with ND96 buffer to appropriate concentrations. The perfusion rate was about 2 mL per minute. The induced-inward current was converted to digital data with the pClamp 10 software (Axon Instruments, Union City, CA, USA) for further analysis.

The current-voltage relationship was measured by changing the holding potential using a ramp protocol. Voltages ranging from −100 mV to +60 mV were applied to injected oocytes to measure the voltage ramp trace. The values were converted using Clampfit 9.0 (Molecular Devices, San Jose, CA, USA).

### 4.7. Data Analysis

For electrophysiological experiments, all values are displayed as mean ± S.E.M. (mean standard error). Concentration-response curve data were analyzed using SigmaPlot 10.0 software. Equation data were obtained using OriginPro 9.0 software (Origin, MA, USA) and fitted using the Hill equation y = V_min_ + (V_max_ − V_min_)*[x]^n^/([IC_50_]^n^ + [x]^n^), where V_max_ and V_min_ were the maximum and minimum current, respectively, x was the concentration of ACh or BA, n was the coefficient of interaction, and IC_50_ was the concentration of BA that produced half the maximum inhibitory effect. *p*-values < 0.05 were considered statistically significant.

## Figures and Tables

**Table 1:** Effects of BA on subunit mutants of α3β4 nicotinic acetylcholine receptor.

Subunit Mutants	I_max_	IC_50_	n_H_
Wild α3 + Wild β4	101.5 ± 5.9	12.8 ± 2.0	0.9 ± 0.1
α3 (L257A) + Wild β4	48.9 ± 2.7	18.6 ± 1.7	1.6 ± 0.1
Wild α3 + β4 (I263A)	33.1 ± 3.3	13.4 ± 1.9	2.7 ± 1.0
α3 (L257A) + β4 (I263A)	7.4	26.2	2

Each current was measured with a holding potential of −80 mV. Values are presented as mean ± SEM (*n* = 7–9/group).

## Data Availability

Data presented in this study are available in the article.

## Sample Availability

Samples of compounds are not available from authors.
